# Non-targeted metabolite profiling reveals substantial equivalence of omega-3 enriched *PfFAD3-1* transgenic soybeans

**DOI:** 10.1080/21645698.2026.2620886

**Published:** 2026-01-29

**Authors:** Eun-Ha Kim, Hanyoung Choi, Hyoun-Min Park, Myeong-Ji Kim, Hajeong Kang, Sang-Gu Lee, Hyun Uk Kim, Young-Soo Chung, Seon-Woo Oh

**Affiliations:** a Biosafety Division, National Institute of Agricultural Sciences, Jeonju, Republic of Korea; b Department of Bioindustry & Bioresource Engineering, Sejong University Seoul, Republic of Korea; c Department of Molecular Genetics, College of Natural Resources and Life Science, Dong-A University Busan, Republic of Korea; d Digital Breeding Convergence Division, National Institute of Agricultural Sciences Jeonju, Republic of Korea

**Keywords:** GM soybean, non-targeted metabolomics, substantial equivalence, ω-3 fatty acid

## Abstract

The overexpression of the *Physaria fendleri* ω-3 fatty acid desaturase (*PfFAD3-1*) in soybean (*Glycine max* (L.) Merr.) substantially increases seed α-linolenic acid (18:3) content. To evaluate the compositional safety of *PfFAD3-1* transgenic soybeans, we conducted a three-year, two-location field trial (Jeonju and Gunwi, Republic of Korea) and applied non-targeted metabolite profiling of polar and lipophilic metabolites. Multivariate analyses (PCA, PLS-DA) revealed that environmental factors (site and year) had a strong effect on seed metabolite composition. Volcano plot and variable importance in projection (VIP) analyses indicated that most metabolite differences between *PfFAD3-1* lines and Kwangan (KA) varied across years and locations, reflecting gene – environment interactions. Importantly, the majority of metabolites in *PfFAD3-1* lines remained within the natural variation range of non-GM reference cultivars, supporting substantial equivalence. The consistent deviation was an increase in β-amyrin, a triterpenoid precursor of soyasaponins. LC – MS analysis further confirmed line- and environment-dependent increases in specific saponins, particularly soyasaponin I. Given the established safety of triterpenoids in soybean and the biological coherence of fatty acid – triterpenoid pathway cross-talk, these findings suggest that β-amyrin accumulation represents an intended metabolic adjustment rather than an unintended effect. Collectively, our results demonstrate that non-targeted metabolomics complements conventional OECD-recommended compositional analyses, providing a robust framework for the safety assessment of nutritionally enhanced GM soybeans.

## 1. Introduction

The global demand for omega-3 long-chain polyunsaturated fatty acids, such as eicosapentaenoic acid (EPA) and docosahexaenoic acid (DHA), continues to rise due to their proven health benefits. However, reliance on marine fish oil is increasingly challenged by sustainability concerns, driving the development of plant-based omega-3 sources.^1,2^ Early metabolic engineering efforts successfully reconstructed the complex ω-3 biosynthetic pathway in model plants and oilseeds.^3,4^ Ruiz-López et al.^5^ demonstrated that heterologous expression of desaturases and elongases could enable the accumulation of EPA and DHA in transgenic oilseeds. Significant progress has been made in *Camelina sativa* and *Brassica napus*, where field-tested events now produce nutritionally relevant levels of these fatty acids and, in the case of DHA canola, have received regulatory approval as novel consumer health products.^6–9^ In parallel with these strategies, α-linolenic acid (ALA, 18:3), the principal plant-derived ω-3 fatty acid, has gained increasing recognition as a nutritionally important target in its own right.^10^ ALA is an essential fatty acid and a major contributor to ω-3 intake in plant-based diets, with documented benefits for cardiovascular health and lipid metabolism.^11^ Substantial increases of ALA in rapeseed, hemp, soybean and rice were achieved through desaturase gene overexpression, omics-guided analyses, and intragenic approaches.^12–17^

The safety evaluation of genetically modified (GM) crops has traditionally relied on targeted compositional analyses under the principle of substantial equivalence.^18,19^ According to this framework, the composition of a GM crop is compared with that of its conventional counterpart and the natural range of variation observed among reference varieties.^20,21^ The specific compositional components are defined by crop-specific OECD consensus documents.^22^ If the GM crop falls within this established range for key nutrients, anti-nutrients, and other relevant components, it is considered as safe and nutritious as its conventional counterpart. This approach provides a scientifically robust baseline for evaluating potential unintended effects while accounting for the inherent variability of plant composition.

Although this approach remains central to regulatory science, it may lack sensitivity to subtle or unintended changes.^23,24^ Omics-based methods, particularly metabolomics, provide broader insights by capturing system-wide metabolic shifts. Several metabolomics-based studies have evaluated compositional equivalence in major GM crops.^25–29^ For example, a metabolomics study of *Bacillus thuringiensis* (Bt) rice revealed significant alterations in amino acids, organic acids, and carbohydrate metabolism, although these changes largely remained within the natural variation of conventional varieties.^30^ Similarly, profiling of Bt maize events indicated differences in lipids, amino acids, and secondary metabolites, but environmental conditions exerted a stronger influence on metabolite variation than the transgenic trait itself.^26^ In late blight – resistant GM potato, comparative profiling detected no biologically relevant differences in sugars, amino acids, or glycoalkaloids, supporting substantial equivalence with conventional cultivars.^31^ Conversely, an integrated proteomic – metabolomic analysis of NK603 glyphosate-tolerant maize reported metabolic disturbances and concluded that the GM line was not substantially equivalent to its isogenic control.^28^ However, this interpretation has been debated due to limitations in experimental design, restricted comparators, and the absence of natural variation.^25,27^ Collectively, these studies illustrate both the utility and the challenges of integrating omics into GM crop risk assessment. In this context, regulatory bodies such as EFSA have outlined roadmaps for integrating omics approaches into compositional assessments.^32–35^ However, ongoing discussions emphasize that key challenges remain unresolved, particularly regarding the choice of appropriate comparators, the strong influence of environmental conditions, and the criteria used to determine biological relevance.^27,36–38^

Soybean is one of important oilseed crops worldwide, providing a major source of protein and polyunsaturated fatty acids for human and animal nutrition. However, conventional soybean oil contains relatively low levels of ALA, limiting its contribution to ω-3 fatty acid intake. Our previous one-year, two-location study demonstrated that *PfFAD3-1* soybeans, which overexpress the *Physaria fendleri* ω-3 fatty acid desaturase gene (*PfFAD3-1*), are substantially equivalent to reference varieties in terms of proximates, minerals, and anti-nutrients.^39^ In this context, the present study provides the first multi-year, multi-location metabolomic and compositional evaluation of *PfFAD3-1* transgenic soybean. By contrasting *PfFAD3-1* lines with their near-isogenic parent and a set of conventional non-GM cultivars, we examine whether the observed compositional changes remain within the spectrum of natural variation. These findings not only strengthen the evidence base for the compositional safety of *PfFAD3-1* soybean but also illustrate the potential of metabolomics as a complementary tool in the regulatory evaluation of genetically engineered crops.

## 2. Materials and Methods

### 2.1. Soybean Samples

Transgenic soybean lines *PfFAD3-1*#10, #11, and #12, carrying the *Physaria FAD3-1* gene under overexpression in the cultivar Kwangan (KA), were used in this study.^16^ These lines have previously been reported to exhibit a marked reduction in linoleic acid (~72%) and a substantial increase in α-linolenic acid (~320%) compared with the non-transgenic parent.^16,39^ Plants were cultivated between 2020 and 2022 in LMO-designated isolation fields located in Jeonju (JJ) (35°83′08.5700″ N, 127°06′62.2900″ E) and Gunwi (GW) (36°11′24.0800″ N, 128°64′16.6600″ E), Republic of Korea. Alongside these lines, the parental cultivar KA and multiple non-GM reference cultivars were cultivated under identical field conditions. In 2020, Daepung-2 (DP-2), Pungsannamul (PSN), and Pungwon (PW) served as controls; Seonpung (SP) was added in 2021; and two black-seeded cultivars, Socheongja (SCJ) and Cheongja-3 (CJ-3), were incorporated in 2022 to capture broader natural variation. Agronomic practices followed the methods of Kim et al.^39^ Seeds were harvested at physiological maturity (R8 stage), pooled, and stored at ambient temperature until analysis. For metabolite extraction, seeds were ground into fine powder and preserved at −80°C.

### 2.2. Extraction and Profiling of Hydrophilic Metabolites

Hydrophilic metabolites were isolated following a previously established approach^40^ with slight modifications. A sample of 100 mg of seed powder was suspended in 1 mL of methanol/water/chloroform (2.5:1:1, v/v/v) containing ribitol (60 µL, 0.2 mg/mL) as an internal standard. Samples were vortexed and incubated in a Thermomixer Compact (Eppendorf, Hamburg, Germany) at 37°C and 1200 rpm. After centrifugation at 16,000 g for 3 min, the aqueous phase (0.8 mL) was collected, diluted with 0.4 mL water, and centrifuged again. The resulting supernatant was evaporated in a centrifugal concentrator (CVE-2000; Eyela, Tokyo, Japan) for 2 h and subsequently freeze-dried overnight. Derivatization was performed in two steps: methoximation with methoxyamine hydrochloride (20 mg/mL in pyridine, 80 µL, 90 min, 30 °C), followed by silylation with 80 µL N-methyl-N-trimethylsilytrifluoroacetamide (MSTFA) for 30 min at 37°C. Gas Chromatography-Time-of- Flight Mass Spectrometry (GC – TOFMS) analysis was carried out using an Agilent 7890A GC system coupled with a Pegasus HT TOF-MS (LECO, St. Joseph, MI, USA). Chromatographic separation was achieved on a 30 m × 0.25 mm i.d. CP-SIL 8 CB column (0.25 µm film thickness, Varian, Palo Alto, CA, USA) under a split ratio of 1:25. The injector was maintained at 230°C with helium as the carrier gas (1.0 mL/min). The oven program started at 80°C (2 min hold), ramped to 320°C at 15 °C/min, and was held for 10 min. The transfer line and ion source were set to 250°C and 200°C, respectively. Mass spectra were recorded from m/z 85–600 with a detector voltage of 1700 V. The ChromaTOF software was used to assist with peak location. Peak identification was confirmed using the NIST Mass Spectral Library (version 11) and authentic reference standards.

### 2.3. Extraction and Profiling of Lipophilic Metabolites

For extraction of lipophilic metabolites was based on Park et al.^41^ A sample of 30 mg of seed powder was mixed with 3 mL ethanol (0.1% ascorbic acid, w/v) and spiked with 5α-cholestane (0.05 mL, 10 µg/mL) as an internal standard. Samples were vortexed, incubated at 85°C for 5 min, and treated with potassium hydroxide (120 µL, 80%) for saponification. After a further 10 min incubation, tubes were cooled on ice, and metabolites were extracted twice with 1.5 mL hexane. Combined extracts were concentrated under reduced pressure, derivatized with pyridine (30 µL) and MSTFA (30 µL) at 60°C for 30 min, and analyzed on an Agilent 7890A GC coupled to a Pegasus HT TOF-MS. Chromatographic separation was performed on a CP-SIL 8 CB column (30 m × 0.25 mm, 0.25 µm). The injector temperature was set at 290°C, and the oven was programmed from 250°C to 290°C at 10°C/min with a final 10 min hold. Spectral acquisition was conducted between m/z 50–800 with ion source and transfer line temperatures at 230°C and 280°C, respectively. Peak identification was confirmed using the NIST Mass Spectral Library (version 11) and authentic reference standards.

### 2.4. Saponin Analysis Using LC – MS

Saponin compounds were analyzed by Liquid Chromatography Mass Spectrometry (LC – MS) using a modified protocol of Gu et al.^42^ Finely ground seed powder (80 mg) was extracted with 80% methanol, and zidovudine was used as the IS. Five replicate extractions were performed per line. Chromatographic separation was achieved on a BEH C18 column (100 × 2.1 mm, 1.7 µm; Waters) using a Waters Acquity UPLC system coupled to a Xevo G2-S QTOF-MS. A 12-min binary gradient (solvent A: water with 0.1% formic acid; solvent B: acetonitrile with 0.1% formic acid) was applied at a flow rate of 0.35 mL/min with the following program: 99% A (1 min), 99–0% A (8 min), 0% A (1 min), 0–99% A (0.5 min), and re-equilibration at 99% A (2.5 min). Mass detection was conducted in negative ESI mode with capillary voltage at 2.5 kV, sampling cone at 40 V, source and desolvation temperatures of 100°C and 200°C, respectively, and desolvation gas flow of 800 L/h. Data were collected from m/z 50–1500 at 0.2 s/scan and processed using UNIFI software with normalization against the internal standard. Compound identification was supported by an in-house library.

### 2.5. Statistical Analysis

Analytes were quantified based on peak area ratios relative to their internal standards. Multivariate analyses, including principal component analysis (PCA) and partial least squares – discriminant analysis (PLS-DA), were performed using MetaboAnalyst 6.0 (https://www.MetaboAnalyst/Canada). Data were subjected to unit-variance scaling before statistical modeling. For pairwise comparisons (KA vs. *PfFAD3-1* lines), Student’s t-tests were applied. Volcano plots were generated in R to visualize differential metabolite abundance.

## 3. Results

### 3.1. Non-Targeted Metabolite Profiles

GC-TOFMS was used to profile of non-targeted polar and lipophilic metabolites. In total, 47 polar metabolites (17 amino acids, 14 organic acids, 9 sugars, 6 sugar alcohols, and 1 amine) and 20 lipophilic metabolites (2 alcohols, 9 fatty alcohols, 3 sterols, 2 triterpenes, 4 vitamins) were identified in soybean seed powder. The corresponding retention times, selected ions for quantification, and their fragment patterns are given in Supplementary Tables S1 and S2. The quantification results of all analyses based on the peak area ratios relative to that of the IS are given in Supplementary Tables S3-S6.

### 3.2. Regional Variation in Metabolite Composition

**Figure 1.**
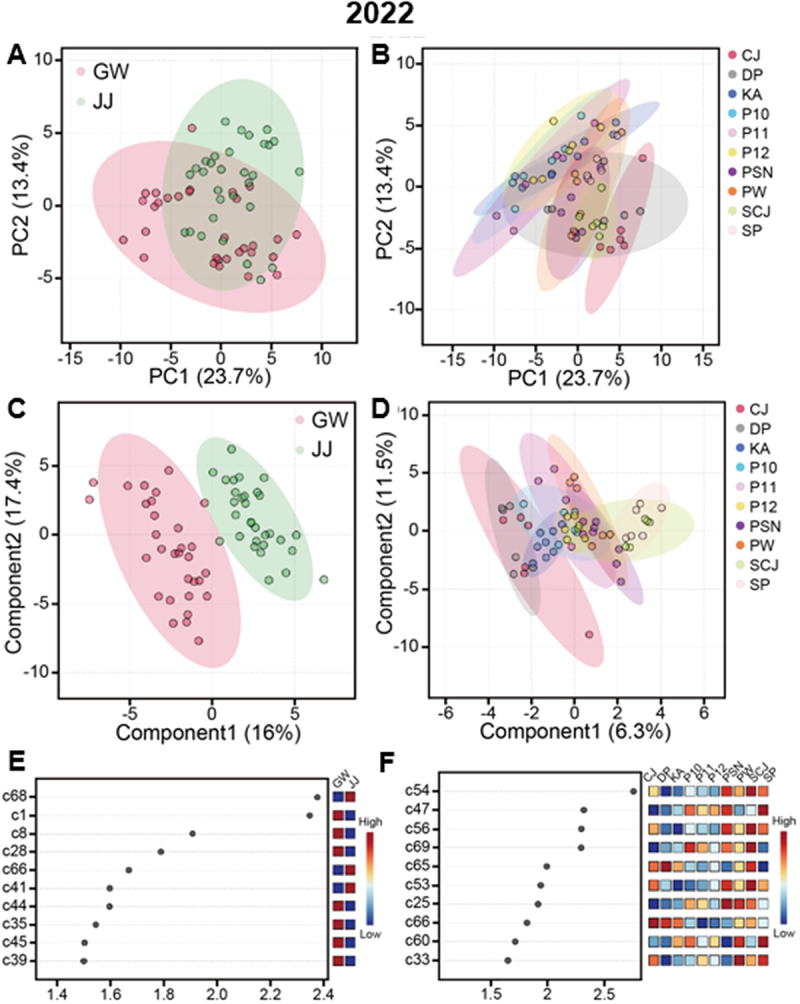
Multivariate analysis of *PfFAD3-1* transgenic soybeans and the parental/reference cultivars in 2022. **(A)** Principal component analysis (PCA) score plot by cultivation site. **(B)** PCA score plot by cultivar. **(C)** Partial least squares–discriminant analysis (PLS-DA) score plot by cultivation site. **(D)** PLS-DA score plot by cultivar. **(E)** Variable importance in projection (VIP) score plot showing the top ten discriminants by cultivation site. **(F)** VIP score plot by soybean cultivar. Metabolite identifiers for (E) and (F) are provided in supplemental table S. P10, P11, and P12 are *PfFAD3-1* lines #10, 11, and #12, respectively.

The data for the 67 metabolites were subjected to PCA and PLS-DA to evaluate the effect of cultivation sites on soybean seed metabolite composition in each cultivation years. In 2020, PCA explained 28.2% (PC1) and 12.1% (PC2) of the total variance, with GW and JJ forming distinct clusters (Supplementary Figure S1a). PLS-DA yielded a clearer discrimination (Supplementary Figure S1b), and VIP analysis identified α-amyrin, galactose, γ-tocopherol, aspartic acid, and stigmasterol as the top discriminants (VIP > 1.8; Supplementary Figure S1c). In 2021, site separation was again observed (PC1 41.9%, PC2 13.1%; Supplementary Figure S2a), with fructose, glucose, ethanolamine, mannitol, and triacontanol (C30-ol) contributing most strongly (VIP > 1.6; Supplementary Figure S2c). In 2022, with additional reference cultivars included, PCA separation was reduced (PC1 23.7%, PC2 13.4%; Figure 1A). Nonetheless, PLS-DA still distinguished GW and JJ samples (Figure 1B), with stigmasterol, lactic acid, ethanolamine, ribose, and brassicasterol identified as key discriminants (VIP > 1.5; Figure 1C). Taken together, these results indicate that although the specific discriminating metabolites varied by year, some metabolites such as stigmasterol, brassicasterol, ethanolamine, glucose, and C30-ol were repeatedly associated with site-specific variation (Supplementary Figure S7), suggesting that these metabolites are sensitive to environment.

### 3.3. Genotypic Variation in Metabolite Composition Separation

PCA and PLS-DA for the 67 metabolites by varieties in each year were performed to evaluate genotypic effects. In 2020, PCA (PC1 28.2%, PC2 12.1%) revealed partial overlap among the parental cultivar, KA and the three *PfFAD3-1* lines (#10, #11, #12), whereas non-GM reference cultivars (DP, PSN, PW) were clearly separated (Supplementary Figure S1b). PLS-DA (C1 39.7%, C2 8.9%) provided a more distinct clustering (Supplementary Figure S1d). VIP analysis identified docosanol (C22-ol), tetracosanol (C24-ol), tryptophan, and triacontanol (C30-ol) as the most discriminating metabolites (VIP > 2.0; Figure 1F). In 2020, a similar pattern was observed, with PCA (PC1 41.9%, PC2 13.1%) showing clearer separation of KA and *PfFAD3-1* lines from reference cultivars, while transgenic lines themselves largely overlapped with KA (Supplementary Figures S2a, S2b). PLS-DA again improved group resolution (Supplementary Figure S2c), and VIP analysis indicated fructose, glucose, ethanolamine, mannitol, and C30-ol as the major discriminants (VIP > 1.6). Notably, fructose and glucose levels were lower in reference cultivars compared with KA and *PfFAD3-1* lines, suggesting that carbohydrate metabolism was a key determinant of varietal clustering in this year. In 2022, with the inclusion of two additional reference cultivars (SCJ, CJ-3), PCA separation between KA/*PfFAD3-1* lines and references was less pronounced (PC1 23.7%, PC2 13.4%; Figure 1A). Nevertheless, PLS-DA revealed improved clustering (Figure 1B), highlighting stigmasterol, lactic acid, ethanolamine, ribose, and brassicasterol as the primary contributors (VIP > 1.5; Figure 1C).

Across all three years, KA and *PfFAD3-1* lines consistently grouped closer to each other than to reference cultivars, reflecting their shared genetic background. However, subtle differences between KA and individual *PfFAD3-1* lines were detectable: *PfFAD3-1*#10 exhibited the largest number of discriminating metabolites in PLS-DA models across all years, indicating greater metabolic responsiveness, while *PfFAD3-1*#12 displayed relatively fewer deviations. Together, these results demonstrate that although transgene insertion did not disrupt the overall genotypic similarity with the parental cultivar, it produced line-specific metabolic adjustments that were stable across environments and years.

### 3.4. Differential Accumulation in PfFad3-1 Lines Relative to KA (Volcano Plot Analysis)

**Figure 2.**
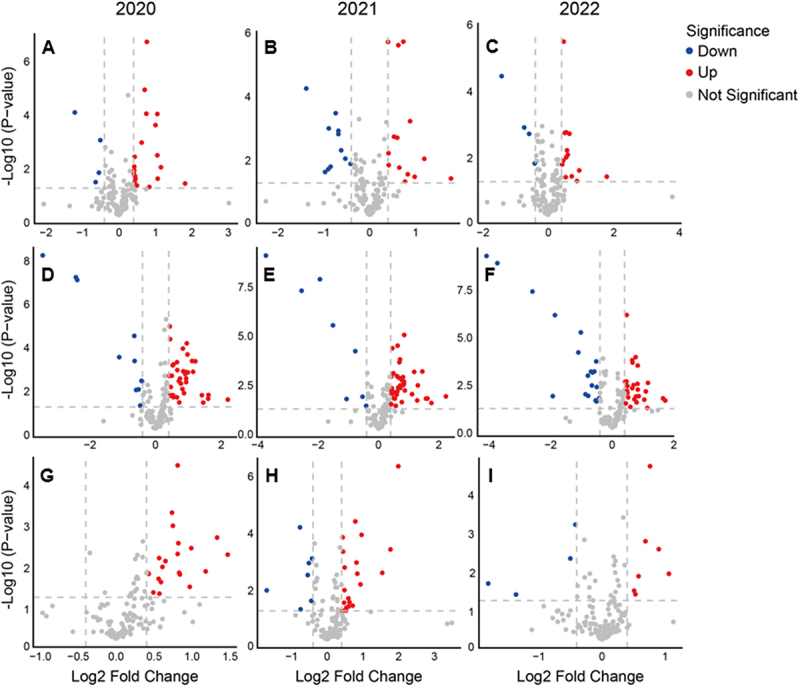
Volcano plots of metabolite differences between *PfFAD3-1* transgenic soybeans and the parental cultivar Kwangan across three years. **(A–C)** lines #10, #11, and #12 in 2020. **(D–F)** lines #10, #11, and #12 in 2021. **(G–I)** lines #10, #11, and #12 in 2022. Each plot shows log_2_ Fold change versus –log_1__0_(p-value). Red, blue, and gray dots represent metabolites significantly increased, decreased, or unchanged, respectively. Vertical dashed gray lines indicate log_2_ Fold change = ±0.4. The horizontal dashed line at –log_1__0_(*p*-value) = 1.30 corresponds to *p* < .05.

Volcano plot analysis integrating both cultivation sites (Figure 2) showed significant changes in metabolite accumulation between KA and *PfFAD3-1* lines (*p* < .05; |log_2_FC| > 0.4) across locations. In 2020, *PfFAD3-1*#10 had 14 increased and 6 decreased metabolites, #11 had 12 increased and 8 decreased, and #12 had 8 increased and 5 decreased. In 2021, the total number of significantly altered metabolites was highest: *PfFAD3-1*#10 (18 up, 10 down), #11 (16 up, 12 down), #12 (11 up, 7 down). In 2022, the numbers were lowest: *PfFAD3-1*#10 (9 up, 4 down), #11 (7 up, 3 down), #12 (6 up, 2 down). Across all years, the number of significantly increased metabolites consistently exceeded those decreased, suggesting a general up-regulation trend in *PfFAD3-1* lines. In the same year, the metabolites that showed significant differences between each *PfFAD3-1* line and KA in GW and JJ were more often different than overlapping. For example, in 2020, *PfFAD3-1*#10 showed seven significantly altered metabolites in GW and sixteen in JJ when compared with KA (Supplementary Table S9-1). Among these, only β-amyrin and ethanolamine were consistently identified at both sites. While β-amyrin was increased relative to KA in both GW (log_2_FC = 0.76) and JJ (log_2_FC = 0.70), ethanolamine displayed divergent patterns, showing a decrease in GW (log_2_FC = −0.64) but an increase in JJ (log_2_FC = 1.05). Similarly, in *PfFAD3-1* soybeans, only a small number of metabolites consistently differed from KW across both sites within the same year, as indicated in bold in the Supplementary Table S9.

### 3.5. Consistently Altered Metabolites Across Years

Metabolites repeatedly altered across years are summarized in Table 1. For *PfFAD3-1*#10, α-tocopherol, β-amyrin, alanine, glycine, serine, and tryptophan in JJ, and lactic acid in GW were consistently different from KA. For *PfFAD3-1*#11, β-amyrin in both sites, lactic acid, and methionine in GW deviated across multiple years. For *PfFAD3-1*#12, β-amyrin was consistently increased across sites, while lactic acid was elevated in GW. To assess the substantial equivalence of GM crops, we compared whether these metabolites in the *PfFAD3-1* lines fell within the minimum and maximum ranges of reference cultivars grown for three years at two sites (Supplementary Tables S9, S10). Importantly, while most metabolites of the *PfFAD3-1* lines remained within the natural variation range of reference cultivars, β-amyrin across all *PfFAD3-1* lines consistently exceeded the maximum value of 1.94 observed in the references (Supplementary Table S11). This result indicates that, with the exception of β-amyrin, the metabolites were substantially equivalent to the natural variation of reference cultivars, and suggests that the change in β-amyrin content is associated with the expression of the *PfFAD3-1* gene.

**Table 1.** Metabolites consistently altered in *PfFAD3-1* transgenic soybean lines across years and locations compared with parental cultivar Kwangan.

Line	Metabolite	Region	2020	2021	2022	Reference range
*PfFAD3-1*#10	α-Tocopherol	Jeonju	3.46 ± 0.46	7.58 ± 0.77	4.02 ± 0.69	1.01 - 9.55
	β-Amyrin	Jeonju	2.02 ± 0.06	2.70 ± 0.35	2.81 ± 0.13	0.71 – 1.94
	Alanine	Jeonju	3.98 ± 0.61	1.90 ± 0.98	0.81 ± 0.20	0.23 – 1.50
	Glycine	Jeonju	1.34 ± 0.09	0.55 ± 0.11	0.29 ± 0.02	0.10 – 0.80
	Serine	Jeonju	0.34 ± 0.05	0.32 ± 0.05	0.16 ± 0.05	0.02 – 0.31
	Tryptophan	Jeonju	2.54 ± 0.26	1.03 ± 0.63	1.13 ± 0.17	0.03 – 3.38
	Lactic acid	Gunwi	0.51 ± 0.05	0.90 ± 0.16	0.72 ± 0.05	0.05 – 0.44
*PfFAD3-1*#11	β-Amyrin	Jeonju	2.06 ± 0.03	2.54 ± 0.30	2.02 ± 0.07	0.71 – 1.94
	β-Amyrin	Gunwi	3.20 ± 0.06	3.23 ± 0.25	2.51 ± 0.23	0.71 – 1.94
	Lactic acid	Gunwi	0.14 ± 0.04	0.28 ± 0.08	0.08 ± 0.02	0.05 – 0.44
	Methionine	Gunwi	0.36 ± 0.03	0.22 ± 0.05	0.12 ± 0.02	0.03 – 0.13
	Arabitol	Gunwi	0.02 ± 0.00	0.07 ± 0.01	0.02 ± 0.02	0.00 – 0.12
	Mannose	Gunwi	0.19 ± 0.01	0.23 ± 0.02	0.30 ± 0.06	0.03 – 0.40
*PfFAD3-1*#12	β-Amyrin	Jeonju	1.83 ± 0.23	2.51 ± 0.04	1.99 ± 0.04	0.71 – 1.94
	β-Amyrin	Gunwi	2.86 ± 0.04	3.36 ± 0.37	2.29 ± 0.20	0.71 – 1.94
	Lactic acid	Gunwi	0.19 ± 0.02	0.36 ± 0.03	0.60 ± 0.10	0.05 – 0.44

Values represent mean ± SD. Reference range indicates minimum – maximum levels observed among non-GM cultivars.

### 3.6. Comparison of β-Amyrin Content in KA and PfFad3-1 Lines

**Figure 3.**
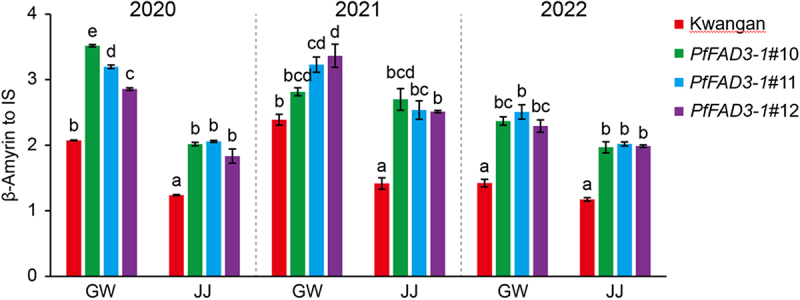
Relative quantification of β-amyrin in *PfFAD3-1* transgenic soybeans and the parental cultivar Kwangan across years (2020–2022) and cultivation sites (GW, JJ). Bars represent mean ± standard deviation (SD; *n* = 3). Statistical significance was determined by two-way ANOVA with Tukey’s post hoc test. Different letters denote significant differences (*p* < .05). GW, Gunwi; JJ, Jeonju.

To investigate the natural variation of β-amyrin under different cultivation environments, we compared its accumulation in KA and the *PfFAD3-1* lines. The β-amyrin content in both KA and the *PfFAD3-1* lines varied depending on cultivation site and year (Figure 3, Supplementary Tables S11, S12). Overall, transgenic lines consistently showed elevated β-amyrin levels compared with KA, with contents being higher in GW than in JJ. These results indicate that β-amyrin accumulation was generally upregulated in *PfFAD3-1* lines across both years and regions. In the same cultivation year, β-amyrin levels in KA were 21–69% higher in GW than in JJ, while *PfFAD3-1*#10 showed 4–74% increase, *PfFAD3-1*#11 a 24–55% increase, and *PfFAD3-1*#12 a 15–56% increase in GW relative to JJ (Supplementary Table S12).

### 3.7. Comparison of Saponin Content in KA and PfFad3-1 Lines

Given that β-amyrin is the precursor of soyasaponins, soyasaponin A1, the LC-MS analysis was performed to quantify saponin profiles (Table 2, Supplementary Table S13). In JJ, *PfFAD3-1*#10 showed no significant differences from KA, whereas *PfFAD3-1*#11 exhibited increased soyasaponin I (+26%, *p* < .05), and *PfFAD3-1*#12 showed elevated soyasaponin Ab (+24%), I (+33%), and Ba (+32%) compared with KA (all *p* < .05). In GW, *PfFAD3-1*#11 showed the most pronounced differences, with significant increases in soyasaponin Ab (+25%), Af (+20%), I (+76%), and γg (+21%) relative to KA. *PfFAD3-1*#12 in GW did not differ significantly from KA. Overall, while the majority of saponins remained within the variation range of KA, soyasaponin I was selectively and substantially elevated in *PfFAD3-1*#11, particularly in GW, indicating metabolic flux from β-amyrin may be preferentially directed toward specific saponin branches under certain environmental conditions.

**Table 2.** Relative quantification of saponins in Kwangan and *PfFAD3-1* transgenic soybean lines cultivated in Jeonju and Gunwi (2022).

Component	Kwangan	*PfFAD3-1*#10	*PfFAD3-1*#11	*PfFAD3-1*#12
Jeonju				
Soyasaponin Ab	5.74 ± 0.32	5.69 ± 0.31	6.04 ± 0.56	7.14 ± 0.98 ^a^
Soyasaponin Af	3.71 ± 0.27	3.85 ± 0.58	3.34 ± 0.30	3.71 ± 0.53
Soyasaponin I	0.61 ± 0.14	0.78 ± 0.15	0.77 ± 0.08 ^a^	0.81 ± 0.13 ^a^
Soyasaponin Ba	0.44 ± 0.02	0.46 ± 0.12	0.46 ± 0.06	0.58 ± 0.09 ^a^
Soyasaponin βa	3.88 ± 0.55	4.25 ± 0.65	4.19 ± 0.44	4.08 ± 0.35
Soyasaponin αg	12.02 ± 2.23	12.96 ± 1.89	10.42 ± 4.27	9.64 ± 3.40
Soyasaponin γg	3.78 ± 0.61	4.45 ± 0.73	4.04 ± 0.39	4.30 ± 0.21
Chromosaponin I	5.38 ± 0.52	6.27 ± 0.92	6.18 ± 0.72	5.78 ± 0.50
Inflasaponin I	1.14 ± 0.18	1.07 ± 0.24	1.12 ± 0.15	1.21 ± 0.13
Gunwi				
Soyasaponin Ab	6.39 ± 0.50	7.35 ± 1.09	8.00 ± 0.75 ^a^	6.62 ± 0.91
Soyasaponin Af	4.42 ± 0.28	4.89 ± 0.73	5.30 ± 0.53 ^a^	4.98 ± 1.09
Soyasaponin I	0.63 ± 0.07	0.86 ± 0.15 ^a^	1.11 ± 0.19 ^a^	0.67 ± 0.30
Soyasaponin Ba	0.45 ± 0.12	0.47 ± 0.31	0.65 ± 0.21	0.49 ± 0.13
Soyasaponin βa	3.89 ± 0.36	3.81 ± 0.48	4.24 ± 0.60	3.97 ± 0.84
Soyasaponin αg	11.48 ± 1.46	12.22 ± 2.81	14.15 ± 2.85	11.76 ± 1.96
Soyasaponin γg	5.14 ± 0.36	5.80 ± 0.53	6.20 ± 0.80 ^a^	6.07 ± 1.28
Chromosaponin I	5.63 ± 0.50	6.00 ± 0.78	5.86 ± 1.90	6.06 ± 1.28
Inflasaponin I	1.42 ± 0.12	1.49 ± 0.18	1.66 ± 0.28	1.64 ± 0.36

Values are mean ± SD (n = 5), normalized to internal standard. Student’s t-test (p<.05) was used to determine significant differences between Kwangan and PfFAD3-1 soybeans, which are indicated by superscript letters.

## 4. Discussion

Previous studies showed that introducing the *PfFAD3-1* gene enhanced the accumulation of α-linolenic acid (18:3) in soybean seeds.^16,39^ This study compared a polar and lipophilic metabolite profiling of *PfFAD3-1* transgenic soybeans with non-transgenic soybeans to assess substantial equivalence. Our analyses revealed that while overall metabolite composition was influenced by environmental variation, specifically the triterpenoid precursor β-amyrin consistently distinguished *PfFAD3-1* lines from the parental cultivar and commercial varieties.

### 4.1. Influence of Environmental and Genotypic Factors

Chemometric methods such as PCA and PLS-DA are useful for classifying compositional data sets from different environments or genotypes.^43–45^ In our study, PCA and PLS-DA score plot results consistently showed clustering of samples according to cultivation site, confirming that environmental factors such as soil, temperature, and precipitation exert strong influence on soybean seed composition.^39,43,46^ Although discriminating metabolites varied annually, stigmasterol, brassicasterol, ethanolamine, and glucose were repeatedly identified as contributors to regional separation by VIP analysis (Figure 2). In PCA and PLS-DA analyses by variety, the *PfFAD3-1* lines clustered very closely with KA, indicating that their metabolite composition was largely similar to that of the parental cultivar due to shared genetic characteristics. However, differences in the degree of overlap and dispersion relative to KA among the individual transgenic lines suggests that unintended metabolic changes may have occurred depending on the insertion site, expression level of the transgene, pleiotropy or somaclonal variation.^47,48^ Random integration of transgenes during plant transformation can influence not only the expression of the foreign gene but also adjacent host genomic regions, leading to unintended molecular variability among independent events.^49^ This interpretation is further supported by the volcano plot results, which showed that the types of metabolites significantly differing between each transgenic line and KA varied depending on cultivation site and year (Figure 2, Supplementary Tables S9, S10). Moreover, even within the same transgenic line, the specific metabolites showing significant differences from KA were not consistent across environments and cultivation years (Supplementary Tables S9, S10). These findings highlight the importance of considering genotype – by environment interactions (G × E) in the compositional assessment of GM crops.^27,43^

### 4.2. Substantial Equivalence to Reference Cultivars

Metabolomics-based assessments across multiple crops consistently demonstrate that most differences observed between GM and non-GM lines are within the natural variation of conventional germplasm.^27,29,36,43,50^ For instance, in wheat engineered to express additional high-molecular-weight glutenin subunit genes, multi-year, multi-site trials showed that environmental factors such as location and year contributed more strongly to metabolite variation than the transgene insertion, with most differences again falling inside the natural range.^43^ In maize overexpressing *Aspergillus niger phyA2* phytase, non-targeted profiling identified nine significantly altered metabolites compared to its isogenic control; however, when variation across 14 conventional maize lines was considered, only four metabolites – directly associated with the engineered phytase pathway – remained outside the natural range.^29^

In line with previous metabolomics-based assessments, our results should be interpreted in the context of natural metabolic variability. Although several metabolites showed significant differences between KA and the *PfFAD3-1* lines (Figure 2, Supplementary Tables S7, S8), the majority of metabolites across three years and two sites fell within the minimum – maximum range of reference cultivars (Table 1, Supplementary Tables S9, S10), thereby supporting the substantial equivalence of *PfFAD3-1* soybeans. Importantly, β-amyrin consistently exceeded the natural variation range observed among non-GM references. While β-amyrin levels in *PfFAD3-1* lines were significantly higher than in KA, the magnitude of these increases was generally comparable to the variation observed across years and locations (Figure 2, Supplementary Table S11). For example, in 2020, β-amyrin levels in *PfFAD3-1*#10 were 63% higher in JJ and 70% higher in GW relative to KA, whereas the difference between GW and JJ for the same line was 74% (Supplementary Table S12). This indicates that both genetic modification and environmental conditions contributed to the observed differences. In contrast, in 2021, β-amyrin in *PfFAD3-1*#10 from JJ increased by 91% relative to KA, exceeding the variability observed between environments (Supplementary Table S11). This suggests that β-amyrin accumulation is dependent not only by genotype and environment individually but also by their interactions (G×E). Importantly, multi-year and multi-location studies remain essential to define the contributions of genotype, environment, and their interactions to compositional variation.

### 4.3. β-Amyrin as a Robust Metabolic Marker of PfFad3-1 Transformation

β-amyrin accumulation in all *PfFAD3-1* lines was reproducibly and consistently elevated relative to KA (Table 1, Figure 2). As β-amyrin is a precursor of soyasaponins and a central metabolite in triterpenoid biosynthesis,^51–55^ its sustained increase is not best interpreted as an unintended perturbation but rather as a biologically coherent outcome of *PfFAD3-1* transformation. The introduction of an ω-3 fatty acid desaturase elevates 18:3, the substrate for jasmonic acid (JA) biosynthesis, thereby suggesting potential cross-talk between fatty acid desaturation and isoprenoid pathways. The JA biosynthetic pathway is highly sensitive to 18:3 availabilities: Arabidopsis *fad3 fad7 fad8* triple mutants lacking 18:3 fail to accumulate JA,^56^ whereas overexpression of FAD3 in the endoplasmic reticulum membrane increased the level of 18:3, which in turn accelerated wound-induced JA biosynthesis and enhanced resistance against the chewing herbivores.^57^ Consistent with this, an integrated transcriptomic – metabolomic study in hemp seeds demonstrated that developmental increases in 18:3 were associated with dynamic shifts in JA intermediates.^13^ Since JA is a well-established regulator of triterpenoid and saponin biosynthetic genes,^58,59^ the consistently elevated β-amyrin and soyasaponin levels in *PfFAD3-1* soybeans are most likely associated with enhanced JA-mediated signaling. Although JA level was not quantified in this study, the observed metabolite profiles suggest that the *PfFAD3-1* gene may induce a coordinated metabolic adjustment linking lipid metabolism to triterpenoid biosynthesis. Further direct measurements will be necessary to validate this mechanistic hypothesis.

Previous non-targeted metabolomics studies in GM crop safety assessment indicate that metabolite-level differences between transgenic and conventional plants are generally interpretable within established metabolic pathways.^60,61^ For instance, in stacked transgenic maize carrying insecticidal cry genes and a glyphosate-tolerance *epsps* gene, non-targeted metabolomics demonstrated that the number and magnitude of differentially accumulated metabolites were limited and fell within the range of natural variation observed among non-GM maize varieties. Pathway-focused analysis further showed that these differences were primarily associated with the shikimate pathway directly linked to the introduced EPSPS trait.^60^ Similarly, GC – MS – based metabolite profiling of a protopanaxadiol-enriched transgenic rice line identified differences in tocopherols, tocotrienols, and phytosterols relative to non-GM rice, which were attributed to competition for shared biosynthetic precursors in the mevalonate pathway rather than to unexpected metabolic perturbations.^61^ Collectively, these findings demonstrate that non-targeted metabolomics is effective in detecting metabolite differences while enabling biologically meaningful, pathway-based interpretation, supporting its value as a complementary tool in GMO safety assessment.

β-amyrin and soyasaponins are endogenous triterpenoid metabolites naturally present in soybean seeds and soybean-derived foods.^62,63^ β-amyrin functions as a key biosynthetic precursor for soyasaponins,^51,55,64^ which represent a major class of legume-specific secondary metabolites with a long history of dietary exposure.^65–67^ Experimental animal studies provide supportive evidence that these compounds do not elicit overt toxicological concerns at biologically relevant exposure levels.^68,69^ In particular, administration of α- and β-amyrin in rodent models of acute inflammation, such as L-arginine-induced pancreatitis, has been shown to attenuate tissue injury, suppress inflammatory cytokine production, and reduce oxidative stress markers without observable adverse effects, even at doses substantially exceeding estimated dietary intake levels.^68^ Given the extensive history of human exposure to these metabolites through conventional soybean consumption, the modest increases in β-amyrin and selected soyasaponins observed in *PfFAD3-1* transgenic soybeans are unlikely to pose nutritional or toxicological risks.

## 5. Conclusion

Non-targeted metabolite profiling demonstrated that *PfFAD3-1* soybeans are substantially equivalent to their parental cultivar, KA and non-GM references, with the exception of β-amyrin. The consistent elevation of β-amyrin, a precursor of soyasaponins, is likely associated with altered fatty acid desaturation and may be linked to JA – mediated signaling pathways. While this interpretation remains hypothetical, it provides a plausible explanation for the observed metabolic changes. Importantly, our results show that non-targeted metabolite profiling can complement traditional compositional analyses recommended by OECD guidelines by providing a broader overview of metabolic variation in GM crops. As β-amyrin and its downstream soyasaponins are naturally occurring compounds in soybean and have a long history of safe consumption, the observed changes are unlikely to pose toxicological or nutritional concerns. From a regulatory perspective, this finding supports the conclusion that *PfFAD3-1* soybeans remain substantially equivalent to conventional cultivars. Collectively, this study contributes robust metabolomics data that reinforce the safety assessment of *PfFAD3-1* soybeans and support their evaluation under diverse environmental conditions. The present findings also illustrate that non-targeted metabolomics can provide biologically meaningful, pathway-based insights into metabolic differences between GM and non-GM crops.

## Supplementary Material

Supplementary Information and Tables.xlsx

Supplementary Figure S1.png

Supplementary Figure S2.png

## Data Availability

The original contributions presented in the study are included in the article/Supplemental Material. Further inquiries can be directed to the corresponding author.
