# Supplementary figures and images for: Non-targeted metabolite profiling reveals substantial equivalence of omega-3 enriched PfFAD3-1 transgenic soybeans
Source: GM Crops Food. 2026 Jan 29;17(1):2620886. doi: 10.1080/21645698.2026.2620886 (PMC12867456; doi:10.1080/21645698.2026.2620886)

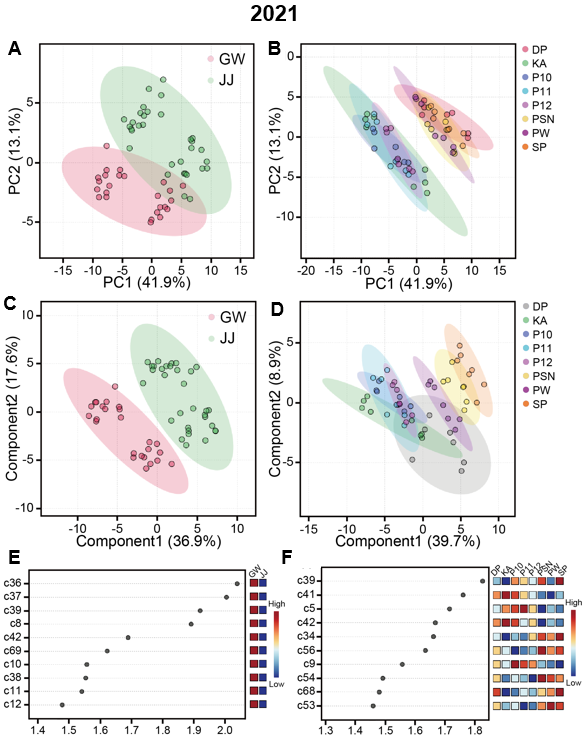

Supplement: Supplementary Figure S1.png [file KGMC_A_2620886_SM7646.png]

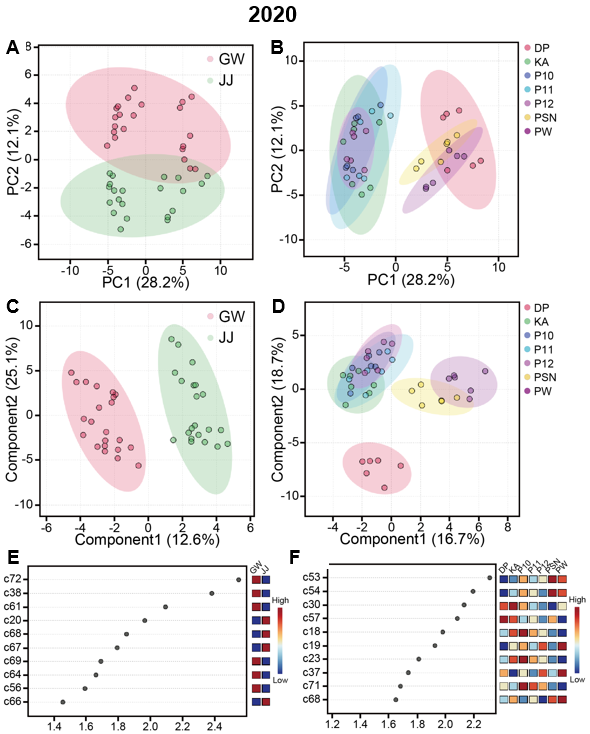

Supplement: Supplementary Figure S2.png [file KGMC_A_2620886_SM7645.png]
